# Impacts of Diet on Reproductive Performance of Captive American Alligators (*Alligator mississippiensis*)

**DOI:** 10.3390/ani13243797

**Published:** 2023-12-08

**Authors:** Ted Joanen, Ashley P. Mikolajczyk, Mark Staton, Josh Kaplan, William E. Holmes, Mark E. Zappi

**Affiliations:** 1 Louisiana Department of Wildlife and Fisheries, Rockefeller Wildlife Refuge, Grand Chenier, LA 70643, USA; 2 Department of Chemical Engineering, University of Louisiana at Lafayette, Lafayette, LA 70504, USA; 3 Energy Institute of Louisiana, University of Louisiana at Lafayette, Lafayette, LA 70503, USA; 4 Mark Staton, LLC, Lafayette, LA 70506, USA; 5 Golden Ranch Farms, LLC, Gheens, LA 70355, USA

**Keywords:** *Alligator mississippiensis*, alligator, captive breeding, nutrition, fertility, hatch rates, early embryonic mortality

## Abstract

**Simple Summary:**

A significant issue in American alligator (*Alligator mississippiensis*) farming is the high rate of early embryonic deaths among captive eggs. Differences in fatty acid composition between wild and captive eggs have been noted, potentially due to maternal diets. A study was conducted at Golden Ranch using captive breeding alligators fed a specialized diet, supplemented with crawfish waste products. Results showed successful reproductive performance. The breeding females consistently achieved high hatch rates, and the resulting hatchlings exhibited comparable strength and size to their wild counterparts. This suggests that *A. mississippiensis* can be farmed effectively using captive breeders raised from artificially incubated wild eggs.

**Abstract:**

Historically, there has been little success with the captive breeding of American alligators (*Alligator mississippiensis*) for both commercial and conservative purposes. This study, conducted at Golden Ranch in Gheens, LA, between 2016 and 2022, utilized a newly formulated commercial feed and practical dietary supplementation (crawfish waste products) to enhance egg production, fertility, and hatch rates. The primary focus of this study was to compare the outcome of this captive breeding program at Golden Ranch with a program conducted at Rockefeller Refuge (RR) between 1979 and 1984. Notable success was achieved in terms of reproductive performance in comparison to the captive breeding program conducted at Rockefeller Refuge. In this study, 16.1 hatchlings were produced per nest on Golden Ranch from captive breeders. Additionally, when wild nests from Golden Ranch were incubated in the same controlled environmental chambers, they produced an average of 16.3 hatchlings per nest. This comparison emphasizes the similarity in egg production between captive-bred *A. mississippiensis* and their wild counterparts. The findings of this study suggest that a closed farming system for *A. mississippiensis* can be established by employing captive breeders derived from artificially incubated wild eggs. Furthermore, American alligators raised in controlled environmental chambers during their initial three years of life demonstrated adaptability to captive conditions and tolerated stocking rates associated with farming conditions and served as breeding stock.

## 1. Background

The potential extinction of numerous crocodilian species can be attributed to factors such as habitat loss and the economic incentive for uncontrolled skin trading. Recognizing the urgency of the situation, the conservation community has turned to captive propagation as an invaluable tool in the endeavor to safeguard these species [1]. This approach is considered preeminent in preventing extinctions, offering two promising mechanisms: the reintroduction of captive-bred specimens into their natural habitats [2] or commercial production to offset the overharvesting of wild populations [3]. 

The establishment of captive populations has demonstrated particular efficacy in mitigating the risk of extinction for species characterized by limited extant populations [2]. Notably, crocodile rearing and farming have played roles in the conservation of species such as the Nile crocodile (*Crocodylus niloticus*) [4], Australian saltwater crocodile (*Crocodylus porosus*) [3], and Philippine crocodile (*Crocodylus mindorensis*) [5].

Captive breeding of critically threatened species would be of great value to conservation efforts. A few studies have reported successful breeding of crocodilian species in captivity [6], but the overall incidence of successful breeding remains relatively rare [7]. Captive breeding efforts have been met with low hatch and fertility rates, early embryonic deaths, or a complete failure to nest. A variety of factors have been advanced to hypothesize results to date including genetic diversity, stress, environmental and behavioral factors, and diet. For example, early embryonic death can be attributed to a variety of factors, including incorrect incubation temperature, low air humidity, poor nutrition of the adult female, a lack of oxygen during incubation process, or external damage during egg transportation [8].

Diet is a critical factor in the health and reproductive success of captive crocodilians, and adequate nutrition is undoubtedly essential to optimize their reproductive capabilities. Whether at zoos or on farms, captive breeding operations must understand and address the unique nutritional requirements of breeding adults. White et al. emphasized that the continued survival of endangered species may depend on captive breeding programs and that successful reproduction depends upon manufactured and/or supplemented diets [9]. In the present study, we report findings of a multi-year study on the effect of diet on the captive breeding of American alligators (*Alligator mississippiensis*) and discuss factors contributing to the reproductive success of this species. 

## 2. Introduction

The successful breeding of American alligators in captivity is relevant to both conservation and commercial interests [10,11]. Considerable resources have been invested by the Louisiana Department of Wildlife and Fisheries (LDWF) in an extensive research program aimed broadly at establishing the feasibility of raising American alligators in captivity for commercial and conservation purposes [11,12,13,14]. In Louisiana, the primary source of eggs for commercial operations is from the harvest of wild eggs, or alligator ranching, while relatively few eggs are produced from captive breeding efforts.

Although developments in captive breeding, egg incubation, and subsequent offspring production have been extensive [11], challenges with sustainability and economics of captive breeding continue. A major setback is the high frequency of early embryonic deaths amongst eggs of captive breeders, causing major expenses to alligator farmers [15]. 

American alligator eggs contain a large yolk rich in lipids, which supplies the developing embryo with the major proportion of its energy requirements and the nutrition necessary for developing the essential tissue components of the embryo [16]. Previous studies have evidenced a range of both total lipid and fatty acid compositional changes that accompany incubation and lipid utilization for the normal development of the embryo [15,16]. When comparing the fatty acid compositions of wild egg yolks to that of captive eggs, there are significant differences in the longer chain, polyunsaturated fatty acids (PUFAs). There were higher levels of unsaturated C18s in the captive eggs compared to the wild and markedly lower levels of the C20 and C22 PUFAs. The dissimilarity between the two groups is perhaps due to the difference in maternal diet [17]. These variations in fatty acid compositions between wild and captive eggs may also have significant implications for the development and viability of captive-bred hatchlings. 

The evidence that the fatty acid compositions of the yolk lipids of both wild and captive American alligators closely reflect those of the respective maternal diets suggest a simple, practical means of rectifying these compositional differences by dietary supplementation, with possible greater hatchabilities of their eggs [17]. Dietary supplementation studies have been performed in American alligators to deduce correlations between fatty acid supplementation with egg fertility [18] and embryonic development [19]. Their studies reported that animals that received nutrient therapy produced a yield of 5.8 hatchlings per breeding female, as compared to untreated American alligators (controls) producing a yield of 2.3 hatchlings per breeding female. 

Previous studies indicate long-chain fatty acids could influence fertility and hatch rates [16]. Therefore, a study was conducted at Golden Ranch located in Gheens, Louisiana, with the aim to increase reproductive performance and hatchling viability with the addition of specific fatty acids (C20s and C22s) in the form of a commercially produced extruded, pelletized, floating feed as well as practical dietary supplementation. 

The present study compares a captive breeding program conducted at Rockefeller Refuge (RR) between 1979 and 1984 [11] with a captive breeding program conducted on Golden Ranch (GR) between 2016 and 2022 utilizing a newly formulated commercial feed and practical dietary supplementation (crawfish waste products) to improve egg production, fertility, and hatch rates. The females in the RR study and the GR study were similar in female brood stock being hatched from wild eggs and reared entirely in captivity in controlled environmental chambers for the first 3 years of life, then stocked in similarly constructed outside breeder pens at age 3. 

## 3. Materials and Methods

### 3.1. Description of the Study Area

Golden Ranch is located three kilometers south of the town of Gheens in Lafourche Parish and approximately 48.3 km southwest of New Orleans, Louisiana. The property is under private ownership and is approximately 21,000 hectares in size. Originally, the agriculture lands were developed for sugar cane production; however, today, the entire property is managed for wildlife and only a small fragment of sugar cane production remains. Golden Ranch has been in the wild American alligator harvest program for many years. The harvest and quotas for wild American alligators and eggs are determined annually by the Louisiana Department of Wildlife and Fisheries. 

The annual mean temperature for the coastal region is 20 °C with extreme lows from −6 °C to −3 °C and extreme highs from 38 °C to 39 °C. The normal tide from mean low to mean high is approximately 45.72 cm. Due to its proximity to the Gulf of Mexico, Golden Ranch is subject to the damaging hurricane windstorm’s tides. Annual rainfall is approximately 152.4 cm. The average growing season for vegetation is 300 days [20]. 

Wildlife such as whitetail deer (*Odocoileus virginianus*), American alligators (*Alligator mississippiensis*), nutria (*Myocastor coypus*), muskrat (*Ondatra zibethicus*), racoons (*Procyon lotor*), otter (*Lutra canadensis*), mink (*Mustela vison*), and many species of wading birds occupy the area year around while thousands of migrating waterfowl use the coastal marshes on Golden Ranch during their winter migration. 

The inactive delta marshes of Golden Ranch are largely made up of freshwater marshes with a mean average salinity of less than 1 ppt. The dominant emergent vegetation is maidencane (*Panicum hemitomon*), followed by bull tongue (*Sagitaria lancifolia*), cattail (*Typha* spp.), and bull whip (*Scirpus* sp.). Aquatic species such as pond weed (*Potamogeton* sp.), American lotus (*Nelumbo lutea*), bladderwort (*Utricularia* sp.), and floating aquatics such as duckweed (*Lemna minor*) and water hyacinths (*Eichhornia crassipes*) occupy the area [20].

**Scheme 1:**
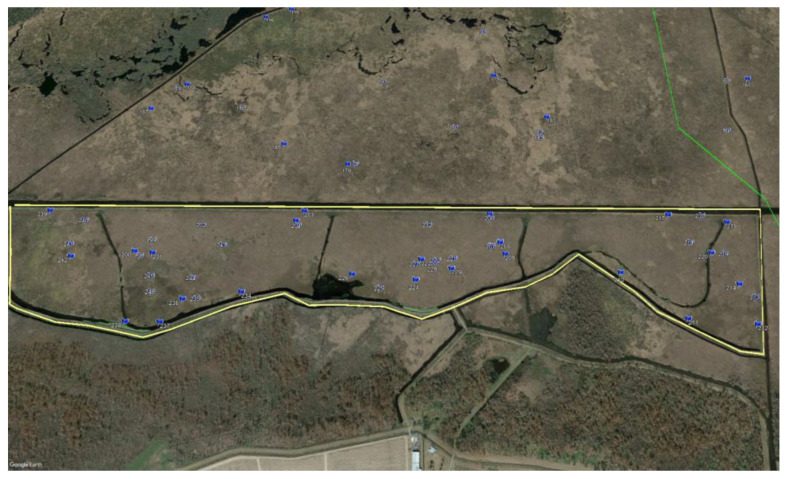
The 121.4 ha pen for captive alligators constructed in 2015 at Golden Ranch in Gheens, Louisiana, with GPS location of nests.

In 2015, a 121.4 ha breeding pen was constructed in a wetland composed primarily of maidencane on Golden Ranch (GR). Canals were dug around the inside of the pen along with small ditches spaced throughout the pen approximately 1.83 m. A land/water ratio of 70/30 was designed to assimilate natural nesting conditions, similar to those described by Joanen and McNease [11] (Scheme 1). Access throughout the pen was by boat. Airboats were utilized for feeding and egg collections. Outboards were used earlier in the study; however, this method was abandoned due to the potential harm to the animals by the underwater propellers. 

### 3.2. Animals

American alligators used in the Golden Ranch study were young alligators (4–6 years old) purchased from a licensed alligator farmer in Louisiana. Eggs were collected from wild nests and the young were raised entirely in captivity for the first three years of life in controlled environmental chambers as described by Joanen and McNease [11]. At this age group, many of the animals were in the 1.2–1.93 m size class.

Approximately 900 American alligators were selected as potential breeders and moved to Golden Ranch in 2016 and released into the outside, fenced breeding pen. Approximately 271 males averaging 1.93 m in length and 618 females averaging 1.73 m in length were stocked in the pen in 2016. Previous studies found that American alligators raised in captivity their entire lives would tolerate higher stocking densities as long as suitable habitat requirements were available for both sexes [11].

### 3.3. Feeds and Feeding

Prior to the development of the test feed, the diet of the American alligators stocked in the fenced breeding pen consisted of the same pelletized feed fed (45% protein, 14% commercial poultry fat) to grow out animals housed in the controlled environmental chambers. 

The test diet was an extruded pellet, manufactured by Cargill, Inc. in Franklin, LA, which contained 45% protein and 14% total fat. To reach this level of fat, poultry and fish oil was added in a 3:2 ratio. High levels of selenium, vitamin E and antioxidants were added as preventatives against oxidation of long-chain fatty acids. 

Fish oil was added to increase the content of the targeted long-chain fatty acids. However, long-chain fatty acids are known to have a short shelf life [21,22], and when fed, only a small fraction of the original amount remained in the pelletized feed. To supplement this short fall, crawfish waste products (*Procambarus clarkii*) were obtained from a commercial seafood processing shed. Food habitat studies examining diets for wild American alligators found crawfish to be an important food source during spring and early summer [23].

Waste products included the heads, part of the hypothalamus, and tails excluding the meat. Prior to processing, live *P. clarkii* were steamed for 5.5 min at 82.2 °C then chilled 5.5 min at 21.1 °C [24]. 

The test diet was fed beginning in 2017 and continued throughout the 2021 breeding season. Crawfish waste products were fed beginning in 2019 and continued throughout the 2021 breeding season. *P. clarkii* is a seasonal crop in Louisiana; therefore, the crawfish waste products were fed over a period of eight weeks each year beginning in mid-April, continuing through May, and ending at the end of the second week in June. 

The test diet and crawfish waste product were discontinued during the 2022 breeding season due to costs along with the time and distance traveled to obtain the crawfish waste products. Beginning in the 2022 season, the diet for captive alligators at Golden Ranch consisted of a dry pelletized floating feed, similar to the diets of the young American alligators held in controlled environmental chambers. 

Twelve feeding sites were established throughout the pen to help disperse the alligators, and these sites were usually near a basking area adjacent to the water’s edge. Feeding began in March of each year and was terminated in late October. Feeding methods as described by Joanen and McNease [12,25] and McNease and Joanen [26] were followed. As the animals aged, careful attention to the amount of food fed on a weekly basis was adhered to so as to avoid obesity. Prior studies suggest that this problem could contribute to a reduction in nesting and egg laying [11]. A feeding rate of 5–6% body weight per week was adhered to during the summer. On average, approximately 910 kg of pellets and 454 kg of crawfish waste products per week were offered during the summer, when most food was consumed.

### 3.4. Egg Collection

Nests were located in early June via helicopter and marked with a Global Positioning System (GPS) shortly after egg laying (Scheme 1). Statistics recorded included the total number of nests, the number of nests with eggs, and total eggs per nest. The numbers of infertile eggs and fertile eggs were recorded. Random eggs were collected for laboratory analysis and all the fertile eggs were artificially incubated at Golden Ranch. 

After hatching, hatchling yields were recorded. At the end of the incubation period, all unhatched eggs were opened, and embryonic deaths were recorded along with the age of the dead embryo. Age was determined by the opaque band adopted from Ferguson [27].

### 3.5. Laboratory Methods

Eggs selected for laboratory analysis by year are shown in Table 1. Wild eggs were collected, except for in 2017, for comparison purposes. In 2015 and 2016, wild eggs (control groups) were collected from Rockefeller Refuge and 2018 and later wild eggs were collected from Golden Ranch (Table 1). After collection, eggs were transported immediately to the Department of Chemical Engineering at the University of Louisiana at Lafayette. The length, width, and weight of the egg was determined. Further analyses were conducted concerning fatty acid analysis of egg yolks and will be discussed in a future publication [28]. 

Fatty acid analysis of crawfish, both fresh and steamed, was performed to validate the presence of long-chain fatty acids. Total lipids were extracted using the Folch method [29]. After extraction, lipids were determined gravimetrically, and lipids were methylated utilizing 2% (*v*/*v*) acidified methanol to convert fatty acids into fatty acid methyl esters (FAMES). The FAMES were extracted using toluene and analyzed by GC-MS equipped with a FAMEWAX column. All fatty acid methyl esters were calibrated using the Marine Oil FAME Mix (100 mg) Standard by Restek^®^ (Bellefonte, PA, USA). 

Eggshells were cleared of any remaining albumen and dried in an oven at low temperatures for 24 h. Eggshells were broken into small pieces and ten random-thickness measurements were performed using a digital micrometer.

## 4. Results

### 4.1. Fatty Acid Analysis of Crawfish

**Figure 1:**
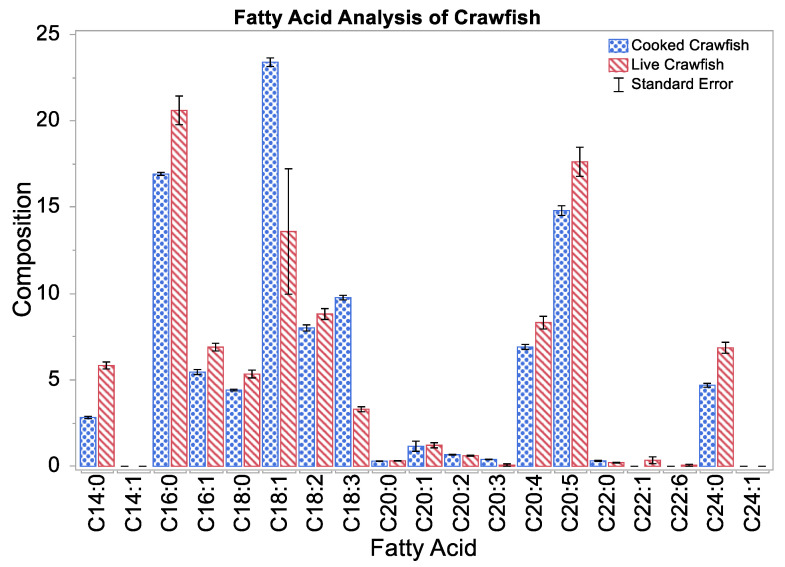
Fatty acid analysis of crawfish and crawfish waste products.

Chemical analysis of the crawfish waste products revealed relatively high concentrations of the desired long-chain fatty acids, except for that of C22:6 (Docosahexaenoic acid) (Figure 1). The total fat content, on a dry weight basis, was measured at 5.75%. 

### 4.2. Age at First Nesting

The age at first nesting for captive American alligators housed and reared in controlled environmental chambers for the first three years of life and then placed in outside pens was 5 years and 10 months, similar to the Rockefeller Refuge study [11]. The age of sexual maturity reported for Louisiana American alligators held in semi-natural outside pens was 9 years and 10 months [13,30]. 

### 4.3. Time of Nesting

The time of egg laying was found to differ for captive American alligators as compared to their wild counterparts. Captive females nested approximately 2 weeks later than their wild counterparts with only a fence separating the two groups. In order to evaluate the differences in timing of egg laying between captive breeders and the wild population, water temperatures in both locations were investigated.

**Scheme 2:**
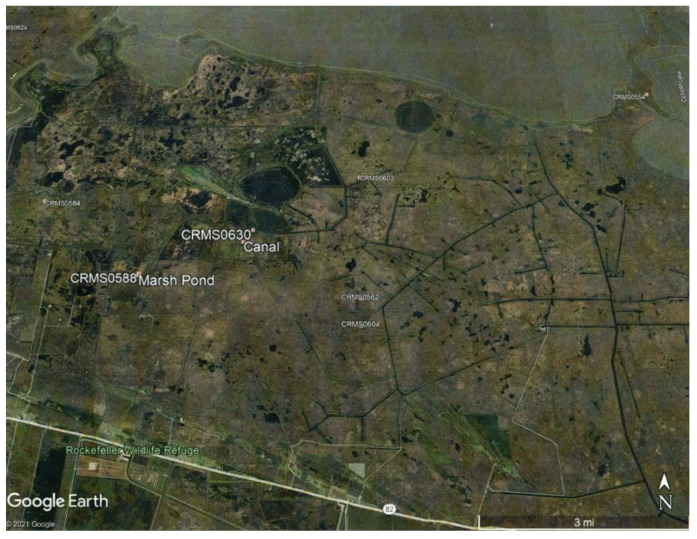
Locations of CPRA 2021 water temperature sites north of Rockefeller Refuge in Grand Chenier, Louisiana.

Marsh water temperature recorders maintained throughout the Louisiana Coastal marshes by the Coastal Protection and Restoration Authority 2021 (CPRA) of Louisiana were analyzed and later compared to the time of nesting. Two sites were selected adjacent to Rockefeller Refuge (Scheme 2). One site was in a shallow marsh pond approximately 15.24–25.4 cm deep and the other was in a 1.83–2.44 m canal used by oil companies for drilling access. Both sites were located in excellent nesting habitats. 

**Figure 2:**
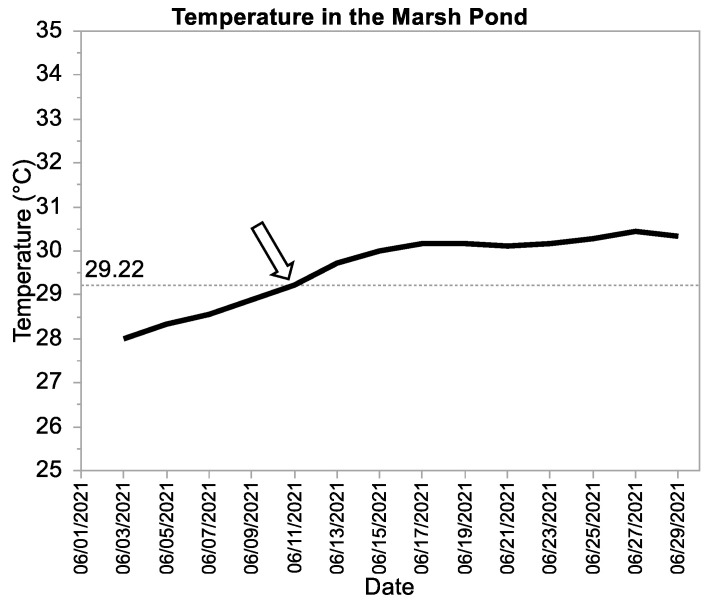
Temperatures in the marsh pond adapted from the Coastal Protection and Restoration Authority (CPRA) of Louisiana 2021, north of Rockefeller Refuge in Grand Chenier, Louisiana. Arrow indicates when the first nest went down.

Spring-time air temperatures collected from Rockefeller Refuge indicated nesting in the wild (egg laying) began on 11 June, peaked on 17 June (when 50% of the nest had eggs), and ended on 23 June in 2021. Analyzing water temperature from CPRA sites just one mile north of Rockefeller Refuge recorded water temperatures in the shallow marsh ponds on 11 June, when the first nest went down in the wild, averaging 29.2 °C (Figure 2).

**Figure 3:**
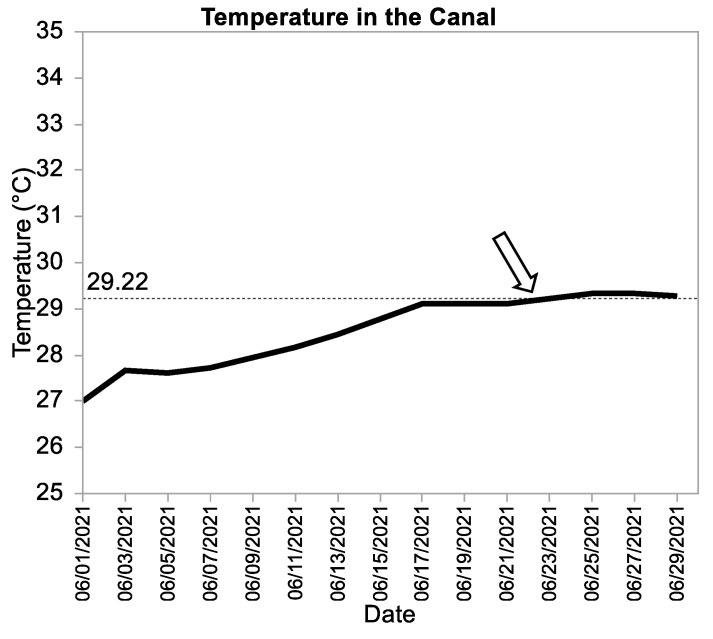
Temperatures in the canal adapted from the Coastal Protection and Restoration Authority (CPRA) of Louisiana 2021), north of RR in Grand Chenier, Louisiana. Arrow indicates when the first nest went down.

The water in the canals took longer to reach the desired nesting temperature levels. The ponds in the captive breeding pens were much deeper (1.83 m) and did not reach 29.2 °C until 12 days later than the shallow (15.24 cm deep) marsh ponds. This could help explain the difference in the timing of nesting between the two groups (Figure 3).

### 4.4. Clutch Size

**Figure 4:**
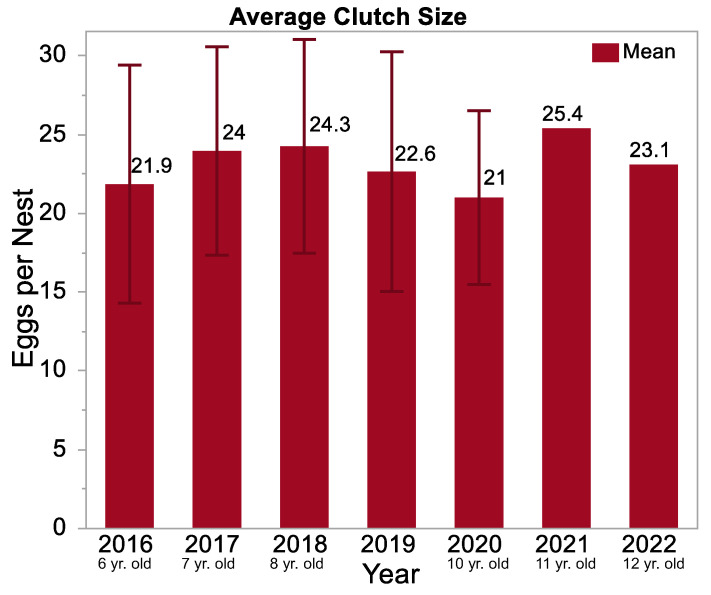
Average clutch size from captive breeders on Golden Ranch (2016–2022).

The clutch size for captive breeding females at Golden Ranch was found to increase with age (Figure 4 and Table 2), similar to what was found in the Rockefeller Refuge study [11]. The first nesting year for captive American alligators on Golden Ranch in 2016 had an average clutch size of 21.9 eggs per nest. The average clutch size for wild nests on Golden Ranch was 25.3 [31]. Penned American alligators on Golden Ranch were found to approach this level of production when they were 8 years old or in their third year of reproduction.

### 4.5. Fertility Rates

**Figure 5:**
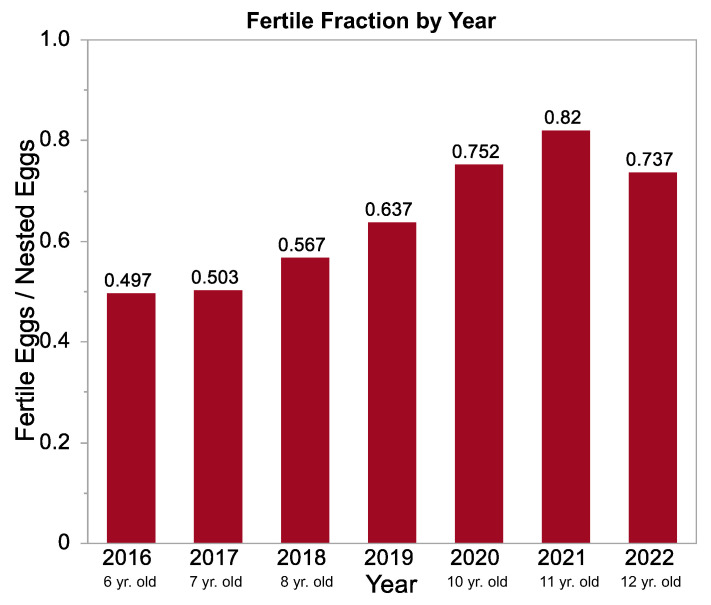
Fraction of fertile eggs defined by total number of fertile eggs/total number of eggs set for captive breeders (2016–2022) on Golden Ranch in Gheens, Louisiana.

Fertility rates were calculated each year as the total number of fertile eggs collected per total number of eggs set for all captive breeders on Golden Ranch. The fertility rates increased as the age of the nesting females increased, similar to what was found in the Rockefeller Refuge study [11] (Table 2 and Figure 5). The fertility rate of 5839 wild eggs examined in Louisiana in 1984 was 95.1% [14]. Dietz and Hines found a fertility rate of 89% in American alligator eggs from Florida in 1980 [32]. Captive breeders on Golden Ranch achieved an 82% fertility rate with 11-year-old alligators. However, there was a drop in fertility to 73.7% in 2022. It is important to note that the test diet and crawfish waste was not administered for the 2022 year. Age of penned females at Golden Ranch could contribute to the difference in fertility as captive females were generally young whereas most of the wild females reported by Dietz and Hines [32] and Joanen and McNease [25] were representative of a mature nesting population.

### 4.6. Hatch Rates

**Figure 6:**
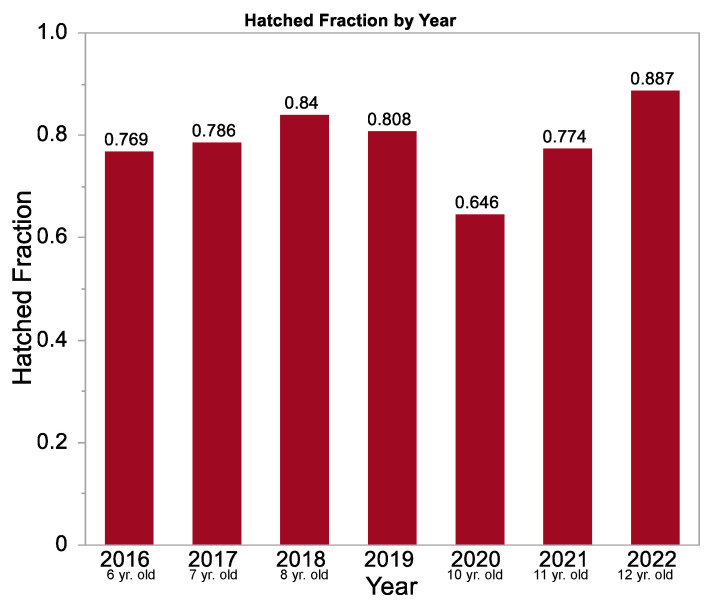
Fraction of fertile *A. mississippiensis* eggs hatched defined by the total number of hatchlings produced/total number of fertile eggs set from captive breeders (2016–2022) on Golden Ranch in Gheens, Louisiana.

Hatch rates were calculated as the total number of hatched eggs per total number of fertile eggs each respective year. Hatch rates for 3963 fertile eggs collected from nests in the pen at Golden Ranch over a 7-year period averaged 78.7% and ranged from a low of 64.6% in 2020 to a high of 88.7% in 2022 (Figure 6). The low years of 2020 and 2021 were associated with flooding conditions on Golden Ranch. Atmospheric low pressure in the Gulf of Mexico generated extremely high tides that inundated the marshes and breeding pen at Golden Ranch which flooded most of the nests and eggs. 

**Figure 7:**
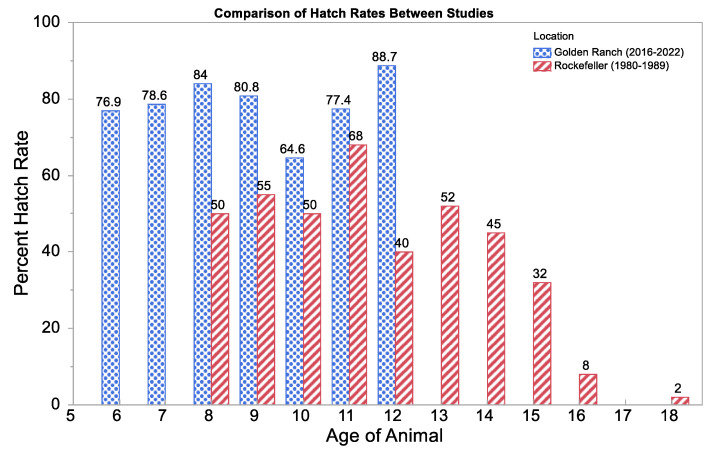
Comparisons of hatch rates of American alligators between Golden Ranch study and Rockefeller Refuge study.

The hatch rate at Rockefeller Refuge began to decline when the alligators approached their twelfth year of life (Figure 7). It is hypothesized that the long-chain fatty acid reserves necessary for embryo growth and development became depleted over this six-year period of captivity. When the Rockefeller Refuge American alligators were approaching 18 years of age, hatch rates were almost reduced to zero [33]. In contrast, the Golden Ranch breeding females maintained an average 78.7% hatch rates during the period under study and recorded their highest hatch rate (88.7%) at age twelve (Figure 7). A two-sample *t*-test of hatch rates from Golden Ranch and Rockefeller Refuge over each study’s respective years was performed. The results of the *t*-test indicated that the hatch rates were statistically different at a 5% significance level, with a *p*-value less than 0.0001.

### 4.7. Embryonic Deaths

**Figure 8:**
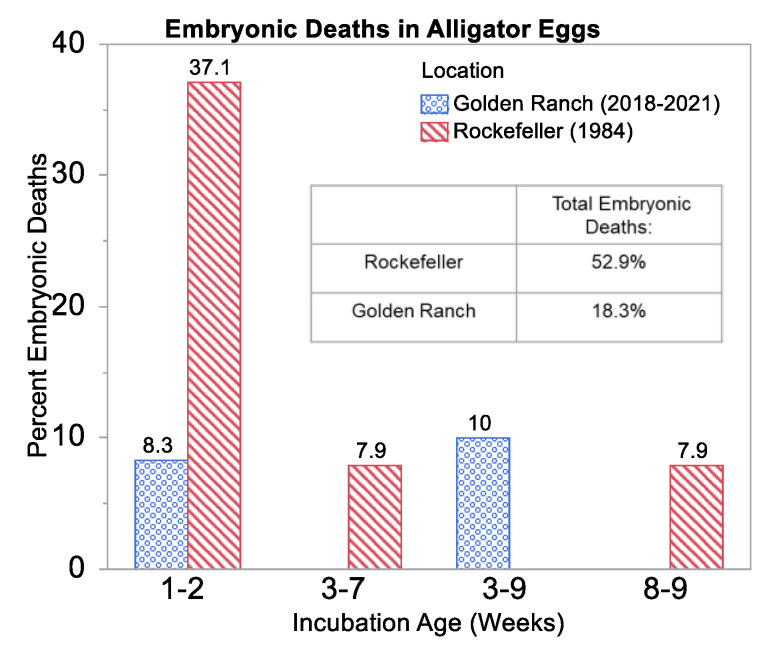
Comparison of early egg mortality of *A. mississippiensis* between Golden Ranch study and Rockefeller Refuge study.

Diets fed to captive breeders on Golden Ranch appear to have reduced early egg mortality (EED) to 8.3% within the first two weeks of egg incubation. Embryonic deaths recorded during the entire 65-day incubation period tallied 18.3% (Figure 8). A comparison of Golden Ranch early embryonic deaths (1–2 weeks) with similar data collected at Rockefeller Refuge in 1978 showed significant differences. In the Rockefeller Refuge study, early embryonic death (1–2 weeks) was recorded to be as high as 37.1% and as high as 52.9% for the entire incubation period [11]. 

### 4.8. Hatchling Production

**Figure 9:**
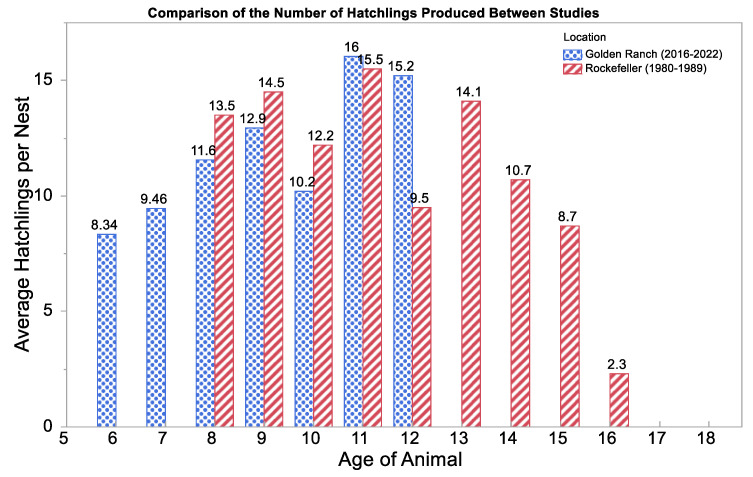
Comparisons of average hatchling per nest of American alligators between this study at Golden Ranch (GR) and the study performed at Rockefeller Refuge (RR).

The number of hatchlings produced per nest for the Rockefeller Refuge study and the Golden Ranch study are presented in Figure 9. Hatchlings produced in the Golden Ranch study ranged from a low of 8.34 hatchlings/nest to a high of 16.1 hatchlings/nest (Table 2 and Figure 9) and generally increased over the seven-year period. Eggs collected from the wild on Golden Ranch and incubated in controlled environmental chambers produced an average of 16.3 hatchlings per nest [34].

### 4.9. Egg Morphology

American alligator eggs collected on Rockefeller Refuge from captive and wild alligators were analyzed by Wink et al. [35], who showed that the eggshells produced by the pen animals were thicker than the wild eggshells. Their studies found that pen eggs had rough, knobby deposits on the shell, along with fewer open pores on the outer surface of the shells. They suggest that decreased porosity of the alligator eggshell may be associated with early embryonic deaths as this was more prevalent in eggs of captive alligators than wild alligators.

Shirley [36] indicated that eggs produced by captive fish-fed American alligators on Rockefeller Refuge had thicker shells than those produced by captive nutria-fed alligators. However, eggs from captive nutria-fed alligators did not hatch as successfully as those from wild American alligators [11].

**Figure 10:**
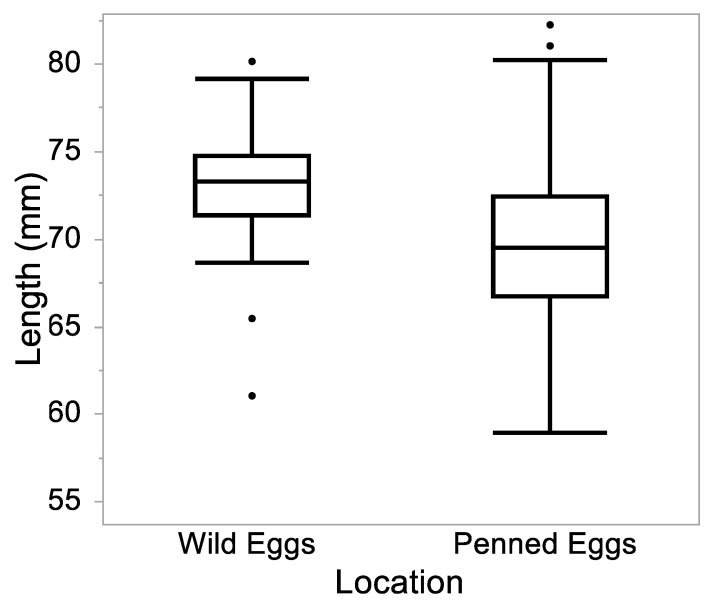
Size comparison of Golden Ranch eggs from captive breeders to that of wild counterparts.

**Figure 11:**
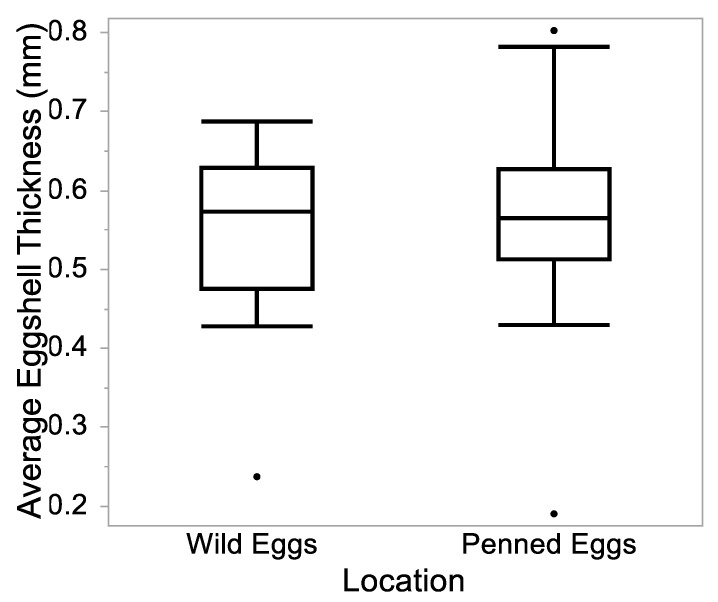
Comparison of average eggshell thickness from captive breeders to that of wild counterparts from Golden Ranch in Gheens, Louisiana (2016–2022).

Measurements (length and width) of the eggs from the pen eggs at Golden Ranch fed the test diet along with crawfish waste products and wild eggs collected on Golden Ranch indicate that the wild eggs were statistically slightly larger (Figure 10 and Table 3). This difference in size could be explained by the relatively young age of the Golden Ranch captive breeders as compared to the Golden Ranch wild population. Comparing shell thickness, the Golden Ranch pen eggshells were found to be slightly thicker than the wild eggs on Golden Ranch, but they were not statistically different (Figure 11 and Table 3). 

The American alligators in the pen on Golden Ranch continue to produce eggs that were comparable to wild eggs in shape, shell thickness, and overall quality. 

## 5. Discussion

This study highlights the importance of both environmental and nutritional factors in the reproductive success of American alligators. Not surprisingly, temperature is a dominant factor in all aspects of the biology of alligators and varies considerably throughout the range of American alligators. In southern Louisiana, Chabreck and Joanen [37] reported approximately seven growing months per year for immature American alligators, and Joanen and McNease [12] reported a similar period for adults. Therefore, for an American alligator to reach sexual maturity in southern Louisiana in 10 years, with 7 growing months per year, a total of about 70 growing months is required. 

Coulson et al. [38] indicated that under laboratory conditions American alligators did not initiate feeding activities at temperatures below 22 °C. According to Fuller et al. [39], the average monthly temperature near the study area in North Carolina was equal to or exceeded 22 °C between May and September. Thus, American alligators in North Carolina only experienced about four active growing months per year in order to reach sexual maturity in the 18–19-year range, equating to a 72–76 growing month requirement [11]. 

Studies by Joanen [40], Fogarty [41], Goodwin and Marion [42], Joanen and McNease [43], Dietz and Hines [32], and Joanen and Merchant [44] indicate that time of nesting (egg laying) is a function of spring time air temperature. Egg deposition in southwest Louisiana occurred in late June and early July when the three-month average (March, April, and May) was 18.3 °C. In contrast, the earliest nesting, late May–early June, occurred during the warmest years when the spring temperatures averaged 21.4 °C [43]. While rainfall did not affect the time of nesting, the amount of spring rain and the associated effect of accrued surface water showed a definite effect on the degree of nesting [44,45].

Time of nesting (egg laying) was found to differ from captive American alligators as compared to their wild counterparts. Captive females nested approximately two weeks later than their wild counterparts even with only a fence separating the two groups. 

The delay in nesting for captive breeders at Rockefeller Refuge was originally hypothesized to be caused by increased stress by the crowded conditions of captivity. Competition for selecting nesting sites and nesting material by captive females could have increased levels of plasma corticosteroids, which could thus delay nest construction and egg laying. However, Elsey et al. [46] found that the captive females had plasma corticosteroid levels that were not significantly different from wild nesting females sampled on the state-owned Rockefeller Refuge. 

Nutrition was also considered a possible explanation for differences between the time of nesting of captive and wild alligators living in close proximity. However, in the present study, we show that adequate nutrition for captive alligators, i.e., maintaining reproductive performance on par with the wild population, can be achieved but did not affect the time of nesting. Captive breeding pens on Rockefeller Refuge were constructed with draglines digging deepwater ponds and canals approximately 1.83–2.13 m deep. Similarly, the pen at Golden Ranch was constructed by digging a canal 1.83–2.13 m deep around the perimeter of the enclosure along with ditches dug throughout the pen, approximately 1.83 m deep connecting ponds, waterways, as well as nesting and feeding sites. The spoil removed to create the canal was allowed to dry and a fence was later placed on the spoil, enclosing the entire area (Scheme 1).

We believe that the data presented here that show the water temperature of the breeding ponds in wild vs. captive circumstances provide a better explanation for the difference in the time of nesting. Ponds within the breeding pens at both Rockefeller and Golden Ranch took longer to reach a desirable temperature for nesting as compared with those in marsh ponds, which were shallower and therefore heated faster. Further studies are needed to confirm this conclusion. 

Clutch size (eggs per nest) increased consistently with age for American alligators in both the Rockefeller Refugee study [11] and the Golden Ranch study (Table 2). The average clutch size was found to differ from southwest Louisiana to southeast Louisiana. The average clutch found in wild nests in southwestern Louisiana was 38.8 eggs [40] while the average clutch found in Southeastern Louisiana was 25.3 eggs [31].

Captive breeders at Golden Ranch reached an average of 25 eggs per nest in their third year of reproduction. At that time, the adult breeders were 8 years old. Adult breeders in the Rockefeller Refuge study reached an average clutch size of 38 eggs per nest in their fourth year of reproduction. At that time, the adult breeders at Rockefeller were 9 years of age.

American alligator farmers who have been collecting eggs on the same property for many years report that the nesting females are much smaller in Southeast Louisiana (nesting females less than 1.83 m in length) as compared to the nesters in Southwest Louisiana. The difference in clutch sizes can be explained by the size differences between the animals in the two groups [44]. Farmers also report that the color is quite different. In southeast Louisiana, females in the fresh maidencane marshes are black in color while the nesters in southwest Louisiana in the intermediate to slightly brackish marshes are more speckled and have a more pronounced yellow strip on their side and tail as compared to their southeastern counterpart [47]. McIlhenny [30] reports that American alligators are susceptible to change in color due to the water they inhabit. The most brilliant-colored alligators are found in the clear salt-water bayous and in salt marshes bordering the Gulf of Mexico.

Throughout the entire study, eggs from the pen on Golden Ranch were comparable to wild eggs in shape, shell thickness, and size. The hatchlings produced were very strong and active, and they began feeding approximately three days after hatching. The hatchlings were mixed with hatchlings produced from eggs collected from wild nests shortly after emerging from the egg with no ill effects [34]. 

The Rockefeller Refuge study reported a major problem in American alligator farming, namely, the observed high frequency of embryonic deaths (ED) among eggs from captive breeding females. Eggs from captive females on Rockefeller Refuge exhibited a 52.9% ED during the entire incubation period whereas the eggs from females in captivity at Golden Ranch had 18.3% ED during entire incubation period (Figure 8). Embryonic deaths from eggs of captive breeders at Golden Ranch showed a reduction in early egg mortality (EED) of 8.3% over a three-year period as compared to a 37.1% EED recorded in the Rockefeller Refuge study. 

This problem worsened over time at Rockefeller Refuge as the hatchability of eggs produced from captive females decreased from year to year [33]. Many of the fertile eggs died in weeks 1–2 of incubation. Also, numerous eggs and in some cases the entire clutch were misshapen, poorly calcified, soft-shelled, crushed, or infertile [33]. In contrast to the results at Golden ranch, hatchlings produced from the Rockefeller Refuge study were weak and had to be maintained in separate pens away from hatchlings produced from wild eggs. Only after several weeks of maintaining the weakened hatchlings at a higher tank temperature did they catch up with the more robust wild-produced hatchlings.

Hatch rates at Golden Ranch were well above Rockefeller Refuge and did not decrease over time as witnessed in the Rockefeller study. Females at Golden Ranch continued to achieve hatch rates acceptable for commercial production despite their young age. The success achieved on Golden Ranch was only slightly below the 91% hatch rate for wild eggs collected and incubated at Rockefeller as reported by Joanen and McNease [48]. The decline in production of live hatchlings in the Rockefeller Refuge study was not immediate and took several years before a serious decline in production was witnessed. This implies that essential fatty acids can take several years to be depleted before significant declines in production are observed, and that in the Golden Ranch study the diet adequately supplemented required fatty acids. This is in agreement with studies by Noble et al. [17] on the problem of early embryonic deaths amongst eggs of captive breeding American alligators. Their studies compared fatty acid compositions of wild eggs to that of captive eggs and found different levels of unsaturated long-chain fatty acids in the captive eggs as compared to wild eggs and suggest that the problems of early embryonic deaths were perhaps due to difference in maternal diets. They concluded that that early embryonic deaths of eggs of captive breeders were due to differences in maternal diets. In the Golden Ranch study, the addition of crawfish waste products (including the heads, part of the hypothalamus, and tail excluding the meat), in addition to the test diet, was found to contain high concentrations of targeted long-chain fatty acids. We believe that the reduction in EED from 37% in the RR study to 8.3% in the Golden Ranch study could only be attributed to diet fed to the breeders. Additionally, the number of hatchlings produced per nest in the Golden Ranch study, and their viability, appear to be directly related to the dietary regime. 

## 6. Conclusions

Results obtained from this study indicate that American alligators can be farmed in a closed system using properly fed breeders, initially produced from eggs that had been artificially incubated and had subsequently been raised in controlled environmental chambers for their first three years of life. These farm-raised alligators have proven to do well in captivity and can tolerate crowded conditions, as would be expected under farming situations [11,33]. It has also been demonstrated that diets have an impact on many aspects of the reproductive performance of these captive breeders, including the clutch size, egg morphology and fatty acid composition, hatch rate, hatchling numbers and viability. 

We believe the findings of this study may extend to other crocodilian species. However, further research needs to be conducted in the formulation of diets for captive breeding crocodilians, with emphasis on the inclusion and protection of long-chain polyunsaturated fatty acids. By addressing these challenges, conservation efforts can not only ensure the long-term sustainability of crocodilian species but also potentially reduce operational costs while simultaneously enhancing both fertility and hatching rates—both critical components of successful conservation strategies. 

## Figures and Tables

**Table 1:** Egg counts for laboratory analysis.

Year	Wild Egg Count	Golden Ranch Pen Eggs
2015	4 eggs × 8 clutches (RR ^1^)	N/A ^3^
2016	4 eggs × 8 clutches (RR)	3 eggs × 24 clutches
2017	None	2 eggs × 16 clutches
2018	2 eggs × 4 clutches (GR ^2^)	2 eggs × 7 clutches
2019	2 eggs × 8 clutches (GR)	2 eggs × 19 clutches
2020	2 eggs × 4 clutches (GR)	2 eggs × 5 clutches

^1^ RR = Rockefeller Refuge. ^2^ GR = Golden Ranch. ^3^ N/A = Not applicable

**Table 2:** Golden Ranch 121.4 ha (300 acre) pen American alligator nesting results (2016–2022).

Year/ Age of Animal	Eggs per Nest	Fertile Rates %	Average Hatchlings per Nest	Hatch Rates %
2016/6 yrs	21.9	49.7	8.34	76.9
2017/7 yrs	24.0	50.3	9.46	78.6
2018/8 yrs	24.3	56.7	11.56	84.0
2019/9 yrs	22.6	61.5	13	80.8
2020/10 yrs	21.0	75.2	10.2	64.6
2021/11 yrs	25.4	82.0	16.1	77.0
2022/12 yrs	23.1	73.7	15.2	89.0

**Table 3:** Egg morphology results for pen and wild eggs from Golden Ranch Gheens, Louisiana (2016–2022).

	Length (mm)		Width (mm)		Eggshell Thickness (mm)	
	Average	Std. Dev.	Average	Std. Dev.	Average	Std. Dev.
Captive Eggs	69.00	4.33	39.20	2.21	0.56	0.08
Wild Eggs	73.08	3.70	42.00	2.37	0.55	0.10

## Data Availability

Data available on request from corresponding author.
